# 膈神经冷冻术在电视辅助胸腔镜肺叶切除术中预防持续性肺漏气的应用

**DOI:** 10.3779/j.issn.1009-3419.2025.102.23

**Published:** 2025-06-30

**Authors:** Qingyu MENG, Yongkun WU, Yufei WANG, Zhanlin GUO

**Affiliations:** ^1^010059 呼和浩特，内蒙古医科大学第一临床医学院（孟庆宇）; ^1^The First Clinical Medical College, Inner Mongolia Medical University, Hohhot 010059, China; ^2^010050 呼和浩特，内蒙古医科大学附属医院胸外科（吴永坤，王宇飞，郭占林）; ^2^Department of Thoracic Surgery, The Affiliated Hospital of Inner Mongolia Medical University, Hohhot 010050, China

**Keywords:** 肺肿瘤, 电视辅助胸腔镜手术, 膈神经冷冻术, 持续性肺漏气, Lung neoplasms, Video-assisted thoracoscopic lobectomy, Phrenic nerve cryotherapy, Prolonged air leak

## Abstract

**背景与目的** 电视辅助胸腔镜手术（video-assisted thoracoscopic surgery, VATS）肺叶切除术是目前治疗肺癌的主要术式，术后持续性肺漏气（prolonged air leak, PAL）是影响VATS肺叶切除术后恢复的重要因素之一。尽管目前临床上有很多防治术后PAL的方法，但术后PAL发生率仍较高。膈神经冷冻术（phrenic nerve cryotherapy, PNC）通过可逆性抑制膈神经功能，诱导膈肌暂时性上抬，从而减小胸腔容积、促进脏壁层胸膜贴合，减少漏气。本研究探讨PNC在VATS肺叶切除术中预防术后PAL的效果。**方法** 纳入2023年6月至2025年1月在内蒙古医科大学附属医院胸外科接受手术的108例符合入组条件的肺癌患者，使用随机数字表法分为对照组（n=54）和试验组（n=54），两组患者均接受VATS肺叶切除及系统性淋巴结清扫术，试验组术中额外行PNC。对两组患者基线特征、术中、术后指标及漏气动态变化进行比较。**结果** 两组患者临床基线资料无统计学差异（*P*>0.05），对照组和试验组患者术后24 h肺漏气发生率（31.5% *vs* 29.6%）和术后PAL发生率（20.4% *vs* 14.8%）均无统计学差异（*P*>0.05）。但对照组从术中漏气测试试验到术后24 h肺漏气过程中，共有8例（32.0%）停止漏气，少于试验组的14例（46.7%）；对照组从术后24 h肺漏气者进展为术后PAL过程中，共有6例（35.3%）停止漏气，少于试验组的8例（50.0%），差异有统计学意义（*P*<0.001）。与对照组相比，试验组术后膈肌上抬更为显著，并在术后3个月恢复，但稍高于术前水平。**结论** PNC联合积极肺修补可作为术中漏气高风险患者的重要干预手段，减少术后PAL发生。

## Abstract (en)

**Background and objective** Video-assisted thoracoscopic surgery (VATS) lobectomy is the primary surgical treatment for lung cancer. A significant factor affecting postoperative recovery is prolonged air leak (PAL). Despite numerous clinical strategies could prevent and manage postoperative PAL, its incidence remains high. Phrenic nerve cryotherapy (PNC) temporarily inhibits phrenic nerve function, causing diaphragm elevation, which reduces thoracic cavity volume, enhances pleural apposition, and mitigates air leakage. This study investigates the efficacy of PNC in preventing postoperative PAL during VATS lobectomy.**Methods** A total of 108 eligible lung cancer patients who underwent surgery at the Department of Thoracic Surgery, The Affiliated Hospital of Inner Mongolia Medical University, from June 2023 to January 2025, were enrolled and randomly assigned to the control group (*n*=54) and the experimental group (*n*=54). The patients in both the two groups received VATS lobectomy and systematic lymph node dissection, with the experimental group also undergoing PNC during the operation. The baseline characteristics, intraoperative, postoperative indicators and dynamic changes in air leakage between the two groups were compared.**Results** The baseline clinical characteristics were comparable between the two groups (*P*>0.05). The incidence of pulmonary air leakage at 24 h after surgery (31.5% *vs* 29.6%) and the incidence of postoperative PAL (20.4% *vs* 14.8%) showed no significant differences between the two groups (*P*>0.05). The intraoperative air leak test to 24 hours after surgery revealed that air leakage ceased in 8 cases (32.0%) in the control group, compared to 14 cases (46.7%) in the experimental group. Moreover, during the progression from air leakage at 24 hours post-surgery to postoperative PAL, air leakage ceased in 6 cases (35.3%) in the control group and 8 cases (50.0%) in the experimental group, with a statistically significant difference (*P*<0.001). Compared to the control group, the patients in the experimental group exhibited more pronounced postoperative diaphragmatic elevation that recovered to a slightly higher than preoperative level by 3 mon after surgery. **Conclusion** The combination of PNC and active lung repair can serve as an important intervention for patients at high risk of intraoperative air leakage, reducing the occurrence of postoperative PAL.

肺癌是我国发病率及死亡率最高的恶性肿瘤^[1]^，手术是治疗肺癌的主要手段，电视辅助胸腔镜手术（video-assisted thoracoscopic surgery, VATS）肺叶切除术凭借切口小、疼痛轻、恢复快的优势，已成为肺癌的标准术式^[2]^。术后持续性肺漏气（prolonged air leak, PAL）是VATS肺叶切除术后的主要并发症之一，不仅延长患者的胸管留置时间，增加医疗费用，还可能导致胸腔感染、呼吸衰竭甚至死亡^[3]^。减少术后PAL的方法包括化学胸膜固定术（chemical pleurodesis, CP）^[4]^、肺切缘覆盖生物胶或纤维蛋白胶^[5]^、数字胸腔引流^[6]^和自体血补片（autologous blood pleurodesis, ABP）^[7]^等，但各有局限性。例如，CP可能诱发全身炎症反应^[8]^；生物材料降解后可引起局部炎症^[9]^，ABP在肺癌手术中应用受限，且可能导致术后发热和脓胸^[7,10]^。近年来，神经功能调控技术为预防术后PAL提供了新方法。膈神经冷冻术（phrenic nerve cryotherapy, PNC）可以使膈神经短时间失活，促使膈肌上抬，缩小胸膜腔、促进胸膜粘连，以减少术后漏气时间^[11]^。本研究旨在探索在VATS肺叶切除术中PNC预防术后PAL的效果。

## 1 资料与方法

### 1.1 研究对象

2023年6月至2025年1月共筛选符合入组条件的肺癌患者108例，使用随机数字表法分为对照组（n=54）和试验组（n=54），两组均接受VATS肺叶切除及系统性淋巴结清扫术，试验组术中额外行PNC。所有入组患者符合纳入和排除标准并签署知情同意。本研究经内蒙古医科大学附属医院医学伦理委员会批准（批准号：No.KY2023010），并在中国临床试验注册中心注册（注册号：ChiCTR2500102181）。

### 1.2 纳入与排除标准

纳入标准：（1）年龄18-75岁，性别不限；（2）术前检查评估为可切除肺癌；（3）手术方式为VATS肺叶切除及系统性淋巴结清扫术，切除一个肺叶或右侧两个肺叶；（4）术后病理诊断为肺癌；（5）患者或其授权委托人术前签署知情同意书。排除标准：（1）行右肺中叶或全肺切除术；（2）中转开胸；（3）术中同时行气管支气管成形术；（4）既往接受过胸腔手术；（5）术中发现患者为闭锁胸腔；（6）患侧或对侧膈神经受损；（7）术后病理非恶性肿瘤。

### 1.3 观察指标和数据收集

收集患者的一般资料、影像学检查及术中、术后的相关指标，具体如下：（1）一般资料：性别、年龄、吸烟史、肺部基础疾病、术前是否新辅助治疗、术后病理学诊断；（2）影像学检查：确定肿瘤部位、肿瘤大小、术前行胸部X线，除外膈神经损伤，并确定术后膈肌上抬及恢复基线；（3）采用肺癌第九版肿瘤原发灶-淋巴结-转移（tumor-node-metastasis, TNM）分期；（4）肺裂发育程度：通过术前薄层胸部计算机断层扫描（computed tomography, CT）及术中情况，按照Craig等^[12]^推荐的方法分为四个等级：①肺裂100%发育为完全肺裂；②>70%肺裂发育；③30%-70%肺裂发育；④<30%肺裂发育；（5）手术时间：从切开皮肤至缝合皮肤结束的时间；（6）术中漏气测试试验：关胸前胸腔试水，麻醉师控制气管内压力为20 cm H_2_O时出现漏气即为术中漏气测试试验阳性，若不漏气即为阴性；（7）术后2 h动脉血氧分压：为气管插管拔除后2 h动脉血氧分压；（8）术后24 h肺漏气：气管插管拔除后24 h开始，直至拔除胸管，观察患者有无肺漏气；（9）术后PAL：定义为术后持续超过5 d的漏气^[13]^。

### 1.4 研究方法

所有患者均接受VATS肺叶切除及系统性淋巴结清扫术，双腔气管插管、全身麻醉后，健侧卧位，于腋中线第7肋间作1.0 cm观察孔置入VATS，腋前线第4或5肋间作3.0 cm左右的操作孔，探查胸腔，闭锁胸腔者剔除试验，观察肺裂发育情况，确定其等级并记录，分离肺裂，依次游离并离断目标肺动脉、静脉及支气管，切除病变肺叶，术中冰冻病理确诊为肺癌者，行系统性淋巴结清扫，切断下肺韧带至下肺静脉水平，肋间神经阻滞，可吸收线缝合肺残面，用温蒸馏水冲洗胸腔试水，尽量修补肺漏气，彻底止血。最后关胸前试水，麻醉师缓慢增加气道压力至20 cm H_2_O，记录患者术中漏气测试试验是否为阳性。放置胸管，关胸，对照组手术结束。试验组于膈肌上方约5 cm处心包表面游离膈神经及其周围脂肪组织，冷冻探头（K320型冷冻手术治疗机，北京库蓝医疗设备有限公司）-65 ^o^C冷冻、持续15 s，冰球形成，然后复温10 s，冰球消失后撤出探头，避免膈神经牵拉；电钩在冷冻点的上缘进行刺激，无膈肌收缩反应，在下缘刺激，能观察到正常的膈肌运动，则认为膈神经冷冻效果满意（图1）。

**图 1:**
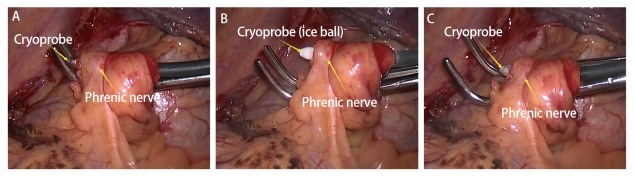
膈神经冷冻术过程。 A：冷冻前：冷冻探头挑起膈神经并远离心包；B：冷冻中：冷冻探头上冰球形成；C：冷冻后：冷冻探头冰球逐渐消失。

### 1.5 统计学方法

采用SPSS 27.0软件进行统计分析。计量资料以均数±标准差或中位数（四分位数间距）表示，组间比较根据数据分布特征选用独立样本t检验或非参数Mann-Whitney U检验；计数资料以例数（百分比）表示，组间差异采用卡方检验或Fisher确切概率法分析。术中漏气测试试验、术后24 h肺漏气和术后PAL通过卡方检验进行组间比较，手术时间、术后2 h动脉血氧分压采用参数或非参数检验。术后24 h肺漏气及术后PAL行多因素Logistic回归分析。所有分析均采用双侧检验，*P*<0.05为差异具有统计学意义。

## 2 结果

### 2.1 两组患者基线特征比较

对照组和试验组在年龄、性别、吸烟史、术前是否行新辅助治疗、病理类型、肿瘤位置、肿瘤直径及TNM分期等方面均无统计学差异（*P*>0.05）（表1）。

**表 1:** 两组患者基线特征差异性比较

Characteristics	Control group (*n*=54)	Experimental group (*n*=54)	*χ*^2^	P
Age, *n* (%)			0.156	0.693
<65 yr	32 (59.3)	34 (63.0)		
≥65 yr	22 (40.7)	20 (37.0)		
Gender, *n* (%)			0.348	0.555
Male	31 (57.4)	34 (63.0)		
Female	23 (42.6)	20 (37.0)		
Smoking, *n* (%)			0.000	>0.999
No	32 (59.3)	32 (59.3)		
Yes	22 (40.7)	22 (40.7)		
Underlying pulmonary diseases, *n* (%)			3.086	0.079
No	27 (50.0)	36 (66.7)		
Yes	27 (50.0)	18 (33.3)		
Preoperative neoadjuvant therapy, *n* (%)			0.121	0.728
No	50 (92.6)	49 (90.7)		
Yes	4 (7.4)	5 (9.3)		
Tumor diameter, *n* (%)			2.611	0.271
<3.0 cm	45 (83.3)	46 (85.2)		
3.0-5.0 cm	9 (16.7)	6 (11.1)		
>5.0 cm	0 (0.0)	2 (3.7)		
Pathological types, *n* (%)			3.012	0.390
Adenocarcinoma	41 (75.9)	40 (74.1)		
Squamous cell carcinoma	8 (14.8)	12 (22.2)		
Small cell lung cancer	3 (5.6)	2 (3.7)		
Carcinoid	2 (3.7)	0 (0.0)		
Upper and lower lungs, *n* (%)			5.101	0.078
Upper lobe	24 (44.4)	27 (50.0)		
Lower lobe	30 (55.6)	23 (42.6)		
Combined pulmonary lobes	0 (0.0)	4 (7.4)		
Left and right lungs, *n* (%)			0.334	0.563
Left lung	24 (44.4)	27 (50.0)		
Right lung	30 (55.6)	27 (50.0)		
Staging, *n* (%)			1.071	0.585
I	38 (70.4)	35 (64.8)		
II	7 (13.0)	11 (20.4)		
III	9 (16.6)	8 (14.8)		

### 2.2 两组患者术中指标比较

对照组和试验组肺裂发育情况均以30%-70%肺裂发育为主，分别为25例（46.3%）、28例（51.9%），其余依次为>70%肺裂发育、<30%肺裂发育和完全肺裂。对照组术中漏气测试试验阳性者较少，共25例（46.3%），而试验组较多，共30例（55.6%）。两组肺裂发育情况、术中漏气测试试验阳性率均无统计学差异（*P*>0.05）。对照组和试验组中位手术时间分别为158、180.5 min，组间无统计学差异（*P*>0.05）（表2）。单因素分析发现肺裂发育情况是影响术中漏气测试试验的重要因素（*P*<0.001），<30%肺裂发育患者术中漏气测试试验阳性率明显高于完全肺裂者，分别为14例（25.5%）、3例（5.4%）。而吸烟史、肺部基础疾病、术前新辅助治疗并不是影响术中漏气测试试验的因素（*P*>0.05）（表3）。

**表 2:** 两组术中指标差异性分析

Variables	Control group (*n*=54)	Experimental group (*n*=54)	*χ*^2^/Z	P
Development status of pulmonary fissures, *n* (%)			0.339	0.953
Complete pulmonary fissure	8 (14.8)	7 (13.0)		
>70% of the pulmonary fissures	12 (22.2)	11 (20.3)		
30%-70% of the pulmonary fissures	25 (46.3)	28 (51.9)		
<30% of the pulmonary fissures	9 (16.7)	8 (14.8)		
Intraoperative air leak test, *n* (%)			0.926	0.336
No	29 (53.7)	24 (44.4)		
Yes	25 (46.3)	30 (55.6)		
Operation time, M (Q_1_, Q_3_), min	158.0 (133.8, 200.0)	180.5 (144.5, 222.5)	-1.322	0.186

M: median.

**表 3:** 术中漏气测试试验因素分析

Variables	No intraoperative air leakage (*n*=53)	Intraoperative air leakage (*n*=55)	*χ*^2^	P
Development status of pulmonary fissures, *n* (%)			15.541	<0.001
Complete pulmonary fissure	12 (22.6)	3 (5.4)		
>70% of the pulmonary fissures	8 (15.1)	15 (27.3)		
30%-70% of the pulmonary fissures	30 (56.6)	23 (41.8)		
<30% of the pulmonary fissures	3 (5.7)	14 (25.5)		
Smoking, *n* (%)			0.054	0.816
No	32 (60.4)	32 (58.2)		
Yes	21 (39.6)	23 (41.8)		
Underlying pulmonary diseases, *n* (%)			1.449	0.229
No	34 (64.2)	29 (52.7)		
Yes	19 (35.8)	26 (47.3)		
Preoperative neoadjuvant therapy, *n* (%)			3.237	0.072
No	46 (86.8)	53 (96.4)		
Yes	7 (13.2)	2 (3.6)		

### 2.3 两组患者术后指标比较

对照组和试验组中位术后2 h动脉血氧分压分别为123、111 mmHg，两组比较无统计学差异（*P*>0.05）。对照组和试验组术后24 h肺漏气分别为17例（31.5%）、16例（29.6%），术后PAL分别为11例（20.4%）、8例（14.8%），两组间术后24 h肺漏气及术后PAL无统计学差异（*P*>0.05）（表4）。单因素分析发现性别（*P*<0.05）、肺裂发育情况（*P*<0.05）、术中漏气测试试验（*P*<0.001）是影响术后24 h肺漏气的相关因素（表5）。肺部基础疾病（*P*<0.05）、肺裂发育情况（*P*<0.001）、术中漏气测试试验（*P*<0.001）是影响术后PAL的因素（表6）。

**表4:** 两组术后指标的差异性分析

Variables	Control group (*n*=54)	Experimental group (*n*=54)	*χ*^2^/Z	P
Oxygen partial pressure at 2 h after surgery, M (Q_1_, Q_3_), mmHg	123 (98.0, 165.0)	111 (91.8, 149.3)	-1.592	0.111
Postoperative air leakage after 24 h, n(%)			0.044	0.835
No	37 (68.5)	38 (70.4)		
Yes	17 (31.5)	16 (29.6)		
Postoperative PAL, *n* (%)			0.575	0.448
No	43 (79.6)	46 (85.2)		
Yes	11 (20.4)	8 (14.8)		

PAL: prolonged air leak.

**表5:** 术后24 h肺漏气发生因素分析

Variables	No postoperative air leakage (*n*=75)	Postoperative air leakage (*n*=33)	*χ*^2^	P
Gender, *n* (%)			4.809	0.028
Male	40 (53.3)	25 (75.8)		
Female	35 (46.7)	8 (24.2)		
Development status of pulmonary fissures, *n* (%)			14.479	0.002
Complete pulmonary fissure	14 (18.7)	1 (3.1)		
>70% of the pulmonary fissures	15 (20.0)	8 (24.2)		
30%-70% of the pulmonary fissures	40 (53.3)	13 (39.4)		
<30% of the pulmonary fissures	6 (8.0)	11 (33.3)		
Smoking, *n* (%)			1.180	0.277
No	47 (62.7)	17 (51.5)		
Yes	28 (37.3)	16 (48.5)		
Underlying pulmonary diseases, *n* (%)			0.909	0.340
No	46 (61.3)	17 (51.5)		
Yes	29 (38.7)	16 (48.5)		
Intraoperative air leak test, *n* (%)			35.180	<0.001
No	51 (68.0)	2 (6.1)		
Yes	24 (32.0)	31 (93.9)		
Preoperative neoadjuvant therapy, *n* (%)			1.749	0.186
No	67 (89.3)	32 (97.0)		
Yes	8 (10.7)	1 (3.0)		

**表6:** 术后PAL因素分析

Variables	No postoperative persistent air leakage (*n*=89)	Postoperative persistent air leakage (*n*=19)	*χ*^2^	P
Gender, *n* (%)			3.387	0.066
Male	50 (56.2)	15 (78.9)		
Female	39 (43.8)	4 (21.1)		
Underlying pulmonary diseases, *n* (%)			4.381	0.036
No	56 (62.9)	7 (36.8)		
Yes	33 (37.1)	12 (63.2)		
Development status of pulmonary fissures, *n* (%)			17.653	<0.001
Complete pulmonary fissure	14 (15.7)	1 (5.3)		
>70% of the pulmonary fissures	20 (22.5)	3 (15.8)		
30%-70% of the pulmonary fissures	47 (52.8)	6 (31.6)		
<30% of the pulmonary fissures	8 (9.0)	9 (47.3)		
Intraoperative air leak test, *n* (%)			13.709	<0.001
No	51 (57.3)	2 (10.5)		
Yes	38 (42.7)	17 (89.5)		
Preoperative neoadjuvant therapy, *n* (%)			0.285	0.594
No	81 (91.0)	18 (94.7)		
Yes	8 (9.0)	1 (5.3)		
Smoking, *n* (%)			0.419	0.517
No	54 (60.7)	10 (52.6)		
Yes	35 (39.3)	9 (47.4)		

将术后24 h肺漏气、术后PAL的相关因素纳入多因素Logistic回归模型分析，结果发现：性别（OR=3.292, 95%CI: 1.028-10.540, *P*<0.05）和术中漏气测试试验（OR=27.054, 95%CI: 5.596-130.792, *P*<0.001）是术后24 h肺漏气的独立影响因素（表7），男性、术中漏气测试试验阳性的患者，其术后24 h肺漏气风险增加。术中漏气测试试验阳性（OR=7.378, 95%CI: 1.458-37.320, *P*<0.05）是术后PAL的独立影响因素（表8），术中漏气测试试验阳性的患者出现术后PAL的风险增加。肺裂发育情况作为一个整体对术后PAL有显著影响（*P*<0.05），但细分后对术后PAL的影响不显著（*P*>0.05）。与对照组相比，试验组术后膈肌上抬更为显著，41例（75.9%）患者在术后3个月内膈肌下降，其余患者在术后6个月内下降，但均稍高于术前水平（图2、3）。

**表7:** 术后24 h肺漏气影响因素的Logistic回归分析

Variables	β	S.E	Wald *χ*^2^	P	OR	95%CI
Constant	-5.100	1.436	12.614	<0.001	0.006	-
Evelopment status of pulmonary fissures*	-	-	4.350	0.226	-	-
>70% of the pulmonary fissures	0.915	1.285	0.507	0.477	2.497	0.201-31.010
30%-70% of the pulmonary fissures	1.153	1.238	0.868	0.351	3.168	0.280-35.846
<30% of the pulmonary fissures	2.304	1.337	2.972	0.085	10.016	0.729-137.530
Intraoperative air leak test (Negative as the reference)	3.298	0.804	16.825	<0.001	27.054	5.596-130.792
Gender (Females as the reference)	1.191	0.594	4.026	0.045	3.292	1.028-10.540

*Complete pulmonary fissure as the reference.

**表8:** 术后PAL影响因素的Logistic回归分析

Variables	β	S.E	Wald *χ*^2^	P	OR	95%CI
Constant	-3.867	1.217	10.106	0.001	0.021	-
Evelopment status of pulmonary fissures	-	-	8.265	0.041	-	-
>70% of the pulmonary fissures	-0.188	1.302	0.021	0.885	0.828	0.065-10.624
30%-70% of the pulmonary fissures	-0.089	1.207	0.005	0.942	0.915	0.086-9.745
<30% of the pulmonary fissures	1.823	1.262	2.087	0.149	6.188	0.522-73.376
Intraoperative air leak test (Negative as the reference)	1.998	0.827	5.838	0.016	7.378	1.458-37.320
Underlying pulmonary diseases (No as the reference)	1.135	0.618	3.370	0.066	3.112	0.926-10.457

**图 2:**
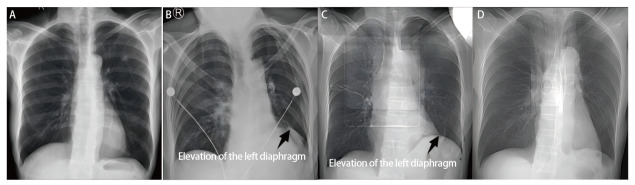
试验组左肺上叶患者术前及术后膈肌变化。 A：术前1天：正常膈肌位置（基线水平）；B：术后1天：胸部X光片显示左侧膈肌上抬（箭头所示）；C：术后1个月：膈肌持续上抬（箭头所示）；D：术后3个月：左侧膈肌恢复。

**图 3:**
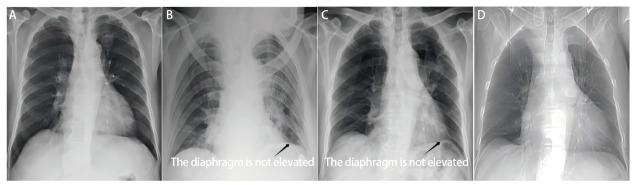
对照组左肺上叶患者术前及术后膈肌变化。 A：术前1天：正常膈肌位置（基线水平）；B：术后1天：胸部X光片显示左侧膈肌未上抬；C：术后1个月：膈肌仍未上抬；D：术后3个月：膈肌未上抬。

### 2.4 两组患者漏气动态变化

对照组和试验组术中漏气测试试验阳性分别为25例（46.3%）、30例（55.6%），术后24 h肺漏气分别为17例（31.5%）、16例（29.6%），两组术后PAL分别为11例（20.4%）、8例（14.8%）；动态观察显示，对照组从术中漏气测试试验到术后24 h肺漏气过程中，共有8例（32.0%）停止漏气，少于试验组的14例（46.7%）。对照组从术后24 h肺漏气者进展为术后PAL过程中，共有6例（35.3%）停止漏气，少于试验组的8例（50.0%）。两组间差异有统计学意义（*P*<0.001）。

## 3 讨论

术后PAL是影响VATS肺叶切除术患者恢复最主要因素之一^[14]^，也是延长患者住院时间的主要因素，严重时可造成肺不张、胸腔感染、胸膜残腔等^[15]^。如何降低肺叶切除术后PAL的发生率及持续时间，一直是肺外科亟需解决的问题。既往在临床工作中，有部分胸外科医师在双肺叶切除或者单肺叶切除加另一个肺叶的局部切除术时，常常会用止血钳夹闭膈神经，以减少术后残腔，并减少术后肺漏气^[16]^。但膈神经钳夹因操作人员使用过程中的不确定性，膈神经钳夹的松紧度难以把控，若钳夹过松，则无法实现膈肌上抬的目标；若钳夹过紧则可能引发膈神经的永久性损伤，进而导致严重的术后并发症。而PNC效果可靠，3个月内膈肌恢复，避免了膈神经永久性损伤的风险^[17]^。因此，本研究观察了VATS肺叶切除术中PNC预防术后PAL的效果。

研究分为对照组和试验组，两组基线数据无统计学差异。研究设计了术中漏气测试试验、术后24 h肺漏气发生率及术后PAL发生率3个指标来观察PNC预防术后肺漏气的效果。

尽管两组术中漏气测试试验阳性率无统计学差异，但试验组术中漏气测试试验阳性者居多，分析发现，试验组肺裂发育不全者居多，与对照组相比有更多的30%-70%肺裂发育者。研究^[18]^显示，在分离发育不全的肺裂时易导致胸膜、肺破裂，增加肺漏气的风险。

本研究单因素分析发现，VATS肺叶切除术后24 h肺漏气的发生与性别、肺裂发育情况及术中漏气测试试验有关，其中，男性可能与其较高的吸烟暴露率有关；术后PAL的发生与肺部基础疾病、肺裂发育情况、术中漏气测试试验有关。而多因素分析发现，不论术后24 h肺漏气的发生还是术后PAL的发生，均与术中漏气测试试验阳性显著有关。这与Wang等^[19]^的研究一致，该研究利用吲哚菁绿近红外荧光技术，术中检测肺漏气点并进行修补，结果表明吲哚菁绿吸入试验可减少术后漏气的发生率。另一项研究^[20]^表明，术中漏气量增多可显著延长术后漏气时间，这说明术中尽力修补肺组织，可有效降低术后24 h肺漏气及术后PAL的发生率。综上，本研究术中未进行更为积极的肺修补，可能是导致术后较高漏气发生率的潜在因素之一。

然而，对照组和试验组的术后24 h肺漏气及术后PAL发生率，均无统计学差异，说明PNC在预防术后24 h肺漏气及降低术后PAL发生率方面无作用。我们的研究结果与既往的一项研究结果^[17]^相反。该研究发现开胸肺叶切除术中PNC可有效减少术后肺漏气发生率，且有统计学差异。开胸肺叶切除术切口疼痛，可导致患者咳嗽咳痰抑制、活动受限，增加肺漏气发生风险^[21]^，从而凸显PNC优势。而VATS手术疼痛程度轻、咳嗽好、下地活动早等，可能降低PNC的效果。同时，在对术中漏气测试试验阳性亚组分析中发现，与试验组相比，对照组术后24 h肺漏气发生率及术后PAL发生率均较高，且有统计学差异。说明PNC可有效降低术中漏气测试试验阳性者发展为术后24 h肺漏气，并减少术后24 h肺漏气转化为术后PAL。

术后24 h内出现肺漏气但24 h后停止的肺漏气未被记录为术后24 h肺漏气。这是因既往研究^[22]^中术后24 h内肺漏气的发生率较高，若将其纳入统计，可能导致术后24 h肺漏气的整体发生率被高估。同时，肺叶切除术后，胸腔容积减少而余肺无法迅速扩张，可能在短时间内形成胸膜残腔，在临床上表现为术后肺漏气，进而影响术后24 h肺漏气发生率的准确性。术后24 h肺漏气的转归主要有两种情况：漏气停止或发展为术后PAL。研究^[23]^显示，80%的术后24 h肺漏气者在未接受任何干预的情况下，其可在发展为术后PAL前自行停止。因此，目前关于术后24 h肺漏气的研究报道相对较少。

目前，关于膈神经可逆性损伤对术后肺功能的影响尚无统一结论。有研究^[24]^发现，膈神经麻痹可降低术后14 d用力肺活量（forced vital capacity, FVC）及第1秒用力呼气量（forced expiratory volume in the first second, FEV_1_）。而另一项研究^[16]^则表明，膈神经钳夹对术后50 d的FVC、FEV_1_无显著影响。这种差异可能与肺功能检测时间点不同有关，术后10 d膈肌位置较术前上抬，导致肺功能指标下降；而到术后50 d，膈肌已基本恢复，肺功能也随之改善。既往研究^[16]^表明，膈神经可逆性损伤对术后早期动脉血氧分压及血氧饱和度无显著影响。这与本研究的结果一致，对照组术后2 h血氧分压稍高于试验组，但无统计学意义，可能是PNC对患者通气功能影响所致，但是与对照组相比，试验组患者术后未见明显气短。

然而，本研究仍存在若干局限性需予以关注：首先，本研究未对术中漏气测试试验、术后24 h肺漏气及术后PAL进行量化，可能存在混杂偏倚。其次，随访过程中未进行肺功能检查，无法评估PNC对远期肺功能的影响。最后，本研究为单中心、前瞻性队列研究，样本量有限，缺乏外部数据验证，存在一定的选择偏倚。因此，未来的研究应对肺漏气进行量化分层，同时术后完善肺功能检查，还需通过多中心随机对照试验扩大样本，以验证结论普适性。

综上所述，PNC可显著降低术中漏气测试试验阳性者发展为术后24 h肺漏气，并减少术后24 h肺漏气转化为术后PAL。因此，在临床中，对于术中漏气测试试验阳性者，应积极进行肺修补，并可考虑应用PNC以减少术后PAL的发生。

**Acknowledgements**

We gratefully acknowledge Rantong BAO from the Center for Clinical Epidemiology Research, Affiliated Hospital of Inner Mongolia Medical University for study design and data analysis.
